# Patient perception about the need for antibiotics after tooth extractions: A cross-sectional study

**DOI:** 10.4317/jced.57938

**Published:** 2021-05-01

**Authors:** Berta Pérez-Amate, Rui Figueiredo, Sergio Cortés-Peral, Alba Sánchez-Torres, Eduard Valmaseda-Castellón

**Affiliations:** 1 DDS. Fellow of the Master degree program in Oral Surgery and Implantology, School of Medicine and Health Sciences, University of Barcelona. Barcelona, Spain; 2 DDS, MS, PhD, Master of Oral Surgery and Implantology. Professor of Oral Surgery and Professor of the Master degree program in Oral Surgery and Implantology, School of Medicine and Health Sciences, University of Barcelona. Researcher at the IDIBELL Institute. Barcelona, Spain; 3 DDS, MS, Master of Oral Surgery and Implantology. Associate Professor of Oral Surgery and Professor of the Master degree program in Oral Surgery and Implantology. School of Medicine and Health Sciences, University of Barcelona. Researcher at the IDIBELL Institute. Barcelona, Spain; 4 DDS, MS, PhD, EBOS. Professor of Oral Surgery and Director of the Master degree program in Oral Surgery and Implantology, School of Medicine and Health Sciences, University of Barcelona. Researcher at the IDIBELL Institute. Barcelona, Spain

## Abstract

**Background:**

Although the current scientific literature does not support the routine use of antibiotics after dental extractions, patients believe that these drugs offer clear benefit during the postoperative period. The main objective of this study was to describe patient perception of the need for antibiotics after routine tooth extraction and to assess knowledge about the benefits and adverse effects of antibiotic therapy.

**Material and Methods:**

A cross-sectional study was carried out. A total of 452 participants requiring tooth extraction and seen in the Dental Clinic of the University of Barcelona (Barcelona, Spain) were given a specific questionnaire on the need for antibiotics after dental treatments or diseases, and on their benefits and adverse effects. Descriptive bivariate and multivariate (logistic regression model) analyses were performed.

**Results:**

Of the 452 participants, 185 (40.9%) were men and 267 (59.1%) were women, with a mean age of 35.2 ± 15.9 years. Most of the patients (76.6%) expected to take antibiotics after tooth extraction. A higher level of education, older age and knowledge about bacterial resistances were inversely correlated to the perceived need for antibiotic treatment (*p*<0.05). According to the respondents, the main advantage of antibiotics was the reduction of infection rates, while the most frequently mentioned adverse events were allergic reactions, diarrhea and nausea or vomiting.

**Conclusions:**

Most patients think that antibiotics are necessary after routine dental extraction to prevent postoperative infection. Younger patients with a low educational level and who are unaware of the problem posed by bacterial resistances seem to be more supportive of antibiotic prophylaxis. Most respondents are familiar with the main benefits and adverse effects of these drugs.

** Key words:**Antibiotic, microbial drug resistance, tooth extraction, oral surgery, survey, postoperative wound infection.

## Introduction

Many professionals routinely prescribe antibiotics after elective surgical procedures (1). However, only patients with a high risk of infection seem to benefit from such prophylactic measures (2,3). The type and dosage of antibiotic is usually decided by the clinician taking into account the available knowledge, though previous experiences are also considered (1,4). However, antibiotic prescription also may be affected by patient expectations and beliefs (5-9).

The widespread use of antibiotics has led to an increase in microbial resistances. The latter are now considered to be a global health problem, since they raise the biological cost for patients (increased morbidity and mortality rates) and constitute an economic burden for national healthcare systems (10-19). Indeed, the World Health Organization (WHO) has considered antibiotic resistance to be a major concern for world health (20). Moreover, the lack of new active drug substances is making bacterial resistance a life-threatening problem, especially in patients with comorbidities. Thus, the use of formulations that incorporate two active drug substances, or the combination of commercially available antibiotics, is a valid alternative in view of the limited discovery of new drugs (4,21).

A Spanish national health survey comprising 19,514 individuals over 16 years of age found 17.7% of the respondents to report self-medication with antibiotics (8). Women, younger patients, higher education levels, toxic habits, the absence of chronic diseases and overall good health were some of the factors associated to antibiotic use without a proper professional recommendation. Another survey in Portugal (region of Algarve) (22) found younger male patients to be more prone to self-medication.

Insufficient knowledge about the indications of antibiotic treatment has also been related to self-medication (7). In this line, some studies have underscored the need to implement education strategies targeted towards the general public in order to increase awareness about correct antibiotic use. This is particularly relevant in reference to frequent viral respiratory illnesses such as colds (7) or following routine surgical procedures (23). In the field of oral surgery, a study conducted in the United States reported that two-thirds of the patients would take antibiotics after routine dental extraction, regardless of their educational level (23).

In view of the above, and given that many patients might believe that antibiotics are needed to prevent complications after simple dental procedures, a survey on patient knowledge about antibiotic use after dental extraction could be useful for establishing educational strategies focusing on the correct use of such drugs. Accordingly, the main objective of the present study was to assess patient perception regarding the need for antibiotics after routine dental extractions in a university dental hospital. The secondary aim was to describe patient knowledge of the main benefits and adverse effects of antibiotic therapy.

## Material and Methods

A cross-sectional study was made involving consecutive patients over 18 years of age seen in the Dental Clinic of the University of Barcelona (Barcelona, Spain) during 2019 for routine tooth extraction. Patients referred to the hospital for other surgical procedures, as well as individuals suffering from mental disorders that might complicate understanding of the study, were excluded. The study followed the Strengthening the Reporting of Observational Studies in Epidemiology (STROBE) statement (24).

The study protocol was approved by the local Institutional Review Board (protocol number 1/2019), and all individuals willing to participate signed the corresponding informed consent. The Declaration of Helsinki guidelines on research involving human subjects were followed throughout the study.

**Figure 1:**
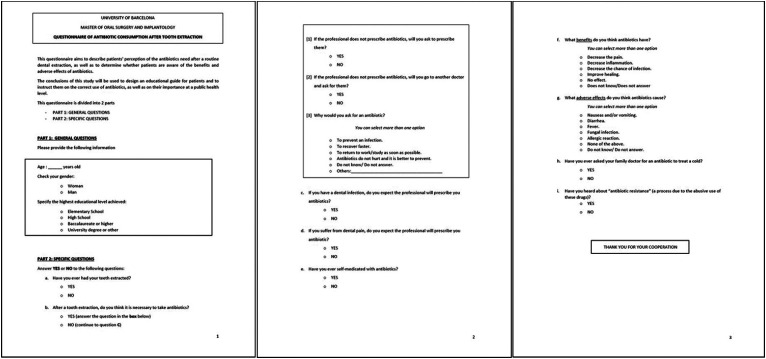
Specific 15-question survey on the need for antibiotic therapy after tooth extraction, to treat infection or tooth pain, on self-medication and the benefits and adverse events of antibiotic treatment.

A specific 15-question survey, adapted from the questionnaire published by Boxx and Laskin (23), was used to explore patient perception about the need for antibiotic therapy after tooth extraction to treat infection or tooth pain, self-medication, and the benefits and adverse effects of such drugs (Fig. 1). The patients were asked to fill the questionnaire while waiting for the appointment, prior to the surgical procedure, and were able to contact the investigators to solve any questions.

Sample size calculation was made using the Stata / IC 15.1 package (StataCorp LLC, Lakeway Drive, TX, USA). A proportion estimation for a finite population of 3000 patients was performed, taking into consideration that 67% of the patients consider it necessary to take an antibiotic after tooth extraction (according to Boxx and Laskin (23)). A total of 452 subjects were required for an absolute accuracy of ± 4% and a 95% confidence level.

Descriptive and bivariate statistics were calculated, and a logistic regression model was generated to detect variables associated to the need for antibiotic treatment, using the Stata / IC 15.1 package (StataCorp LLC, Lakeway Drive, TX, USA).

## Results

A total of 452 patients (185 men (40.9%) and 267 women (59.1%)) with a mean age of 35.2 ± 15.9 years (range 18-83) answered the questionnaire.

Table 1 shows the main clinical features of the sample, as well as the results of the bivariate analysis (associations between the need for antibiotic treatment and the patient characteristics). Younger patients were significantly more inclined to use antibiotics (*p*=0.007), while knowledge about bacterial resistance was associated to a significantly lower perceived need for antibiotic treatment (*p*=0.009).

**Table 1:**
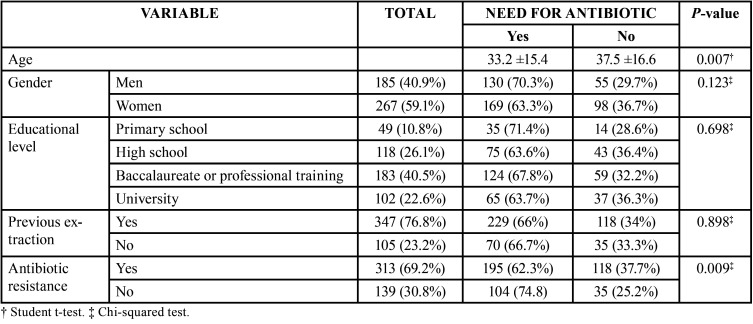
Bivariate analysis showing the relationship between the patient characteristics and the need for antibiotic treatment after tooth extraction.

Regarding specific questions, 347 of the 452 patient (76.6%) expected their dentists to prescribe an antibiotic after tooth extraction. Likewise, almost half of these patients (n=135; 45%) mentioned that if the professional did not prescribe an antibiotic, they would personally request such medication, and 56 subjects (18.7%) would even ask for a professional second opinion. The patients believed antibiotics to be necessary to prevent infections (n=207, 45.8%), to ensure faster recovery (n=80, 17.7%), to return to work or school earlier (n=48, 10.6%), or for no specific reason (n=18, 4%). Forty-one respondents didn’t know or didn’t answer (9.1%).

Dental infections were considered an indication for antibiotic use by 401 patients (88.7%), while 240 participants (53.2%) expected an antibiotic to be prescribed if they suffered toothache. One hundred and one patients (28.9%) had medicated themselves at least once during their lifetime.

**Figure 2:**
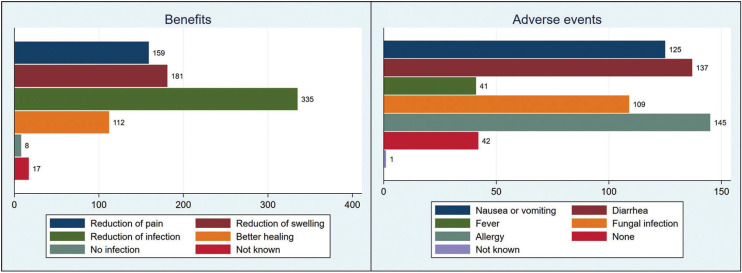
Benefits and adverse events known to the patients.

Figure 2 shows the benefits and adverse effects reported by the patients. Reduction of the infection rate was the most commonly mentioned advantage, while allergic reactions were the more frequently referred adverse event. Nevertheless, 9.3% of the patients considered that antibiotics have no adverse effects at all.

Sixty-five patients (14.4%) reported that they would request their physician to prescribe antibiotics to treat a cold. Lastly, most of the participants (n=313, 69.3%) were familiar with the concept of antibiotic resistance.

The logistic regression model showed a lower educational level, younger age and unawareness of bacterial resistance to be significantly correlated to the perceived need for antibiotic prescription (Table 2).

**Table 2:**
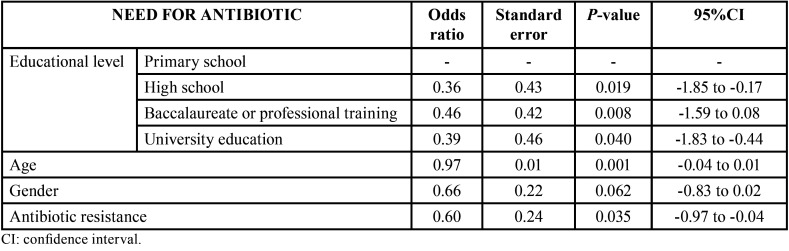
Logistic regression analysis of the need for antibiotic treatment after tooth extraction.

## Discussion

The results of the present survey confirmed the hypothesis that patients have limited awareness concerning antibiotic use after routine tooth extractions. Approximately three out of every four patients expected their dentists to prescribe antibiotic treatment after dental extraction, mainly to prevent postoperative infections. The multivariate analysis showed that older patients with a higher educational level, and with some knowledge about bacterial resistances, made a more conservative judgment regarding the need for antibiotic treatment.

Boxx and Laskin (23), in a similar setting (oral surgery in a university clinic), reported a lower percentage of patients expecting to receive antibiotics (66.7%) compared with our sample (76.6%). Surprisingly, our study presented a lesser proportion of patients (45% versus 70%) that would explicitly request antibiotic treatment if the dentist decided not to prescribe such treatment. In both studies, the prevention of postoperative infections was the main indication for antibiotic treatment. These Figures suggest that the population is not aware of the advantages and disadvantages of antibiotic use for prophylactic purposes. Indeed, most authors agree that routine antibiotic prescription for the prevention of postoperative wound infections in healthy patients is not warranted (6-10). According to a Cochrane review of randomized clinical trials (6), antibiotic use reduced the risk of postoperative infection by 70% when compared to placebo. However, the number needed to treat (NNT) - which quantifies the number of patients to be treated in order to prevent an event from occurring (25) - was found to be 12; while the number needed to harm (NNH) - corresponding to an index of the adverse events associated with a given treatment, meaning the number of patients who should receive one treatment instead of another in order for an additional patient to suffer a harmful event (25) - was shown to be 21. A meta-analysis published in 2007 (26) reported an even lower NNT of 25. These results show that the benefit of routinely prescribing antibiotics does not seem to clearly outweigh the risk of side effects. Furthermore, public health-related issues such as the development of microbial resistances should also be taken into account when conducting a risk-benefit analysis. In our opinion, there is a strong need to implement antibiotic awareness campaigns in order to encourage better practices among the general public (27). In addition, and at a smaller level, it is essential for dental professional to spend more time after tooth extraction explaining to their patients the reasons why antibiotics are not required on a regular basis (23). In this respect, it should be stressed that many professionals are pressured by their patients to prescribe antibiotics.

Self-medication with antibiotics has been a common practice in relation to illnesses of the upper respiratory tract. Interestingly, Grigoryan *et al.* (7), in a study conducted in 19 European countries, found pharyngeal symptoms, bronchitis and tooth or gingival pain to be the most common reasons for self-medication with antibiotics. This could reflect patient knowledge about the use of antibiotics. Our survey included a question related to asking the family physician for antibiotic treatment to treat a cold, and the proportion of patients who would request such treatment was similar to that reported by Boxx and Laskin (23) (14.4% and 15.8%, respectively).

Other publications have focused on overall self-medication. Dental problems are the most common problem for which pharmacists dispense medicines without a medical prescription (2). In fact, studies in Europe have found that it has been possible to purchase antibiotics directly from pharmacies without a prescription, even in the presence of a mandatory statutory requirement (3). Ramalhinho *et al.* (22) showed the level of self-medication in a Portuguese region to be 18.3%. Another study showed that difficulties in accessing healthcare, due to a shortage of physicians or low economic status, might be related to self-medication. Accordingly, patients often may view pharmacists as first-line professionals to solve health problems (3). On the other hand, dispensing antibiotics per package size can produce leftovers, and this has been reported to substantially contribute to self-medication, while dispensing the exact number of antibiotic Tablets could help to reduce this problem (23).

The fact that the patients in our study exhibited a wide age range and a diverse educational background ensures the external validity of the results in the Spanish population. The outcomes of our study underscore the need to develop educational campaigns to increase the awareness of the general population towards the actual needs of antibiotic use, the associated adverse events and, most importantly, to contribute to reduce microbial resistances. Fortunately, public educational campaigns have been shown to be effective in changing attitudes and knowledge about antibiotic use and microbial resistances (27). In addition, and in agreement with Boxx and Laskin (23), we believe it necessary to spend time during the postoperative period to explain to the patients why an antibiotic is not required for their particular situation.

## Conclusions

Most patients believe antibiotic issue to be necessary after routine tooth extraction to prevent postoperative infection. Younger patients, with a lower educational level, and who are not familiar with the concept of bacterial resistance seem to be more prone to self-medication.

Although patient knowledge about the risks and benefits of antibiotic use is generally acceptable, the results of the present survey underscore the need to implement antibiotic awareness campaigns in order to reduce unnecessary antibiotic prescription after tooth extraction.
